# A Review of Methods to Determine Viability, Vitality, and Metabolic Rates in Microbiology

**DOI:** 10.3389/fmicb.2020.547458

**Published:** 2020-11-17

**Authors:** Olivier Braissant, Monika Astasov-Frauenhoffer, Tuomas Waltimo, Gernot Bonkat

**Affiliations:** ^1^Department of Biomedical Engineering, Faculty of Medicine, University of Basel, Allschwil, Switzerland; ^2^Department Research, University Center for Dental Medicine, University of Basel, Basel, Switzerland; ^3^Alta-Uro AG, Basel, Switzerland

**Keywords:** metabolism, viability, assay, redox dyes, electron acceptors, carbon sources

## Abstract

Viability and metabolic assays are commonly used as proxies to assess the overall metabolism of microorganisms. The variety of these assays combined with little information provided by some assay kits or online protocols often leads to mistakes or poor interpretation of the results. In addition, the use of some of these assays is restricted to simple systems (mostly pure cultures), and care must be taken in their application to environmental samples. In this review, the necessary data are compiled to understand the reactions or measurements performed in many of the assays commonly used in various aspects of microbiology. Also, their relationships to each other, as metabolism links many of these assays, resulting in correlations between measured values and parameters, are discussed. Finally, the limitations of these assays are discussed.

## Introduction

Metabolism can be defined as the sum of all reactions in a living organism aimed at maintenance, development, and reproduction (division for microbes). Consequently, measuring all reactions that contribute to metabolism in microbial cells is impossible with current tools even if metabolomics using NMR and mass spectrometry (MS) can provide very valuable snapshots of the metabolites present (Emwas et al., 2013; Alonso et al., 2015; Markley et al., 2017; Zang et al., 2019). In addition, very few methods allow dynamic measurement of metabolism. Therefore, for many purposes ranging from environmental sciences to medical microbiology, microbial metabolism is usually assessed by using proxies that focus on different aspects of the process. Among these so-called metabolic assays, some are nonspecific proxies focusing on various types of metabolism [such as tetrazolium reduction, fluorescein diacetate (FDA) hydrolysis, or calorimetry], while some assays are more focused on and limited to specific types of metabolism. Indeed, one can assess metabolism using electron acceptor consumption (O_2_, NO_3_^–^, SO_4_^2–^, and others) or the production of their reduced form (NO_2_^–^ and H_2_S, for example). Similarly, the consumption of carbon sources and production of by-products (CO_2_, fermentation products, and others) are also valuable tools. As these assays all focus on metabolism, there are correlations between some of them, making comparison or validation possible.

Metabolic assays have been used for a wide variety of applications. For example, in ecological studies, such assays can be used to evaluate the number of active bacteria in a sample (microbial mat, food sample, or other). However, in pharmacological and biotechnological applications, these assays are less often focused on metabolic rates and are mostly used to assess viability to compare the effects of different products on microbial cultures. Similarly, yield and other important information can be gathered from some of these assays or their combinations.

Nevertheless, depending on the intended application parameters, such as volume and sensitivity, the necessary equipment can be a significant factor in the choice of a metabolic assay. For example, metabolic assays with pure cultures can have very different requirements from those with environmental or food samples. Indeed, many of these assays have been devised for pure cultures and are applicable only to such cultures. Many have been modified for applicability to environmental studies, but the resulting limitations are often neglected. In addition, the cost and practicality can also vary a lot, thus influencing potential application. Therefore, it is important to take these parameters into account and review the different assays.

## Confusion Between Amount and Activity: A Brief Warning

Using all the techniques described below, it is crucial to discriminate between activities (i.e., rates) and amounts (values measured at defined time points). In many studies, this difference is neglected, which results in directly linking growth to metabolic activity. Many studies have measured metabolic activity using growth (often measured by a net increase in cell number) as a proxy. Although such approximation can be understood as high metabolic activity is required for growth, it has major flaws. For example, in batch liquid culture, when entering the stationary phase, the cell number still increases while the metabolic activity is often already decreasing. In this context, it is often worth coupling both measures and determining the activity per cell over the course of the culture. Ultimately, at the end of the experiment, usually at the late stationary phase, there is a very large number of cells, but the overall metabolic activity is very low. On the other extreme of the spectrum, it was recently shown that even with no net biomass increase, biofilm could have a very high metabolic activity as measured by calorimetry (Solokhina et al., 2017). This is also true at the community level; for example, in microbial mats, low “apparent” growth rates are observed; however, these microbial structures have been shown to have productivity and turnover equivalent to rain forest (Guerrero and Mas, 1989; Briand et al., 2004). Currently, to assess metabolic activity, it is crucial to measure rates with a sufficient time resolution to allow determination of changes in the rates. It is also crucial that authors pay attention to their use of the terms “metabolism” and “metabolic activity” in their reports and provide a proper explanation of whether rates or amounts (as proxies) are being considered. Overall, the take-home message from this section is that metabolism always implies a rate, and the measure of a proxy in the form of a concentration is still a proxy for a rate.

## Redox Assays

### Tetrazolium Salts

**FIGURE 1:**
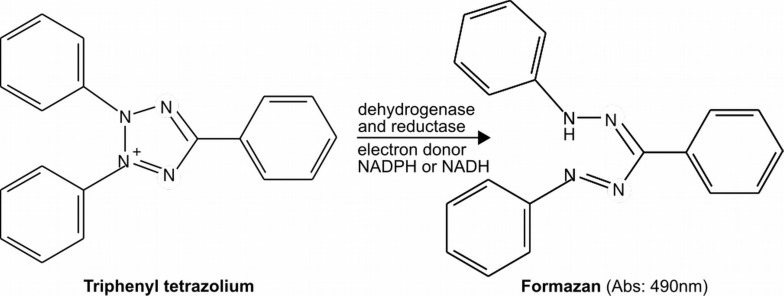
Example of reduction of triphenyl tetrazolium into formazan. Note that many other derivatives of tetrazolium exist (Table 1).

Tetrazolium salts represent a large family of compounds (Table 1) that can be used to measure redox activity in metabolically active cells (Smith and McFeters, 1997; Berridge et al., 2005; Grela et al., 2018; Stockert et al., 2018). In particular, tetrazolium reduction is associated with a functional and active electron transport system (ETS). Colorless tetrazolium salts pass the outer membrane of most tested bacterial cells readily and are then reduced to different red to violet formazan derivatives by reduced nicotinamide adenine dinucleotides (NADH) or their phosphorylated derivative (NADPH)-dependent oxidoreductases and dehydrogenases of metabolically active cells (Figure 1). There are very few data on the ability of archaea to reduce tetrazolium salts, still some pure cultures of *Haloferax volcanii*, *Haloarcula marismortui*, and *Halobacterium sodomense* have been shown to perform such reduction (Oren, 1995). However, it must be noted that tetrazolium might not be able to penetrate a large fraction of fungal and other eukaryotic microbes to reach the mitochondria and thus the active ETS or other enzymes able to reduce it (Kumar and Tarafdar, 2003). Usually, it is considered that sites where reactions occur along metabolic pathways are, in most cases, well-known and that as a consequence of their connection with the ETS, the reactions are correlated with the respiration rate even under anaerobic conditions (Fontvieille et al., 1992). Some of the resulting formazan derivatives are soluble, while others are not. Insoluble formazan compounds formed inside the cells can be extracted with organic solvents (methanol, ethanol, acetone, or other appropriate organic solvent mixtures) to get a quantitative assessment of the amount produced. Several parameters have been considered in optimizing the assay. Among them, final concentration of tetrazolium salts (enzyme saturation vs. dye toxicity), incubation time, added substrates (if any), pH of the assay, and storage conditions of the sample are important. Also, it is important to assess the presence of abiotic reductant, which can trigger abiotic formation of formazan without active biological activity. Several studies have pointed out that flavonoid, plant extract, or another reducing agent might lead to a significant reduction of tetrazolium dyes even in formaldehyde-fixed samples. For this, it is recommended to perform a blank measurement on using a sample fixed by adding formaldehyde to a final concentration of 1.5% (Braissant et al., 2009). Note that a final concentration of formaldehyde up to 4.0% can be used. Similarly, the presence of superoxide might also affect the results (Gong, 1997; Wieder et al., 1998; Créach et al., 2003; Berridge et al., 2005; Wang et al., 2011; Grela et al., 2018).

**TABLE 1:** Summary of physicochemical properties of commercially available tetrazolium-based dyes.

Short name	Full name	Tetrazolium solubility	Absorb. coef. (reduced formazan) cm^–1^⋅M^–1^	Redox intermediate	Formazan solubility
TTC	Triphenyl tetrazolium chloride	50 mg/ml	14,320 (sigma)	Not required	Insoluble
MTT	Thiazolyl blue tetrazolium bromide	5 mg/ml	13,000–16,900 (578 nm)	Not required	Insoluble
INT	Iodonitrotetrazolium chloride	4 mg/ml	12,000 (480–490 nm)	Not required	Insoluble
CTC	5-Cyano-2,3-di-(p-tolyl)tetrazolium chloride	15 mg/ml	NA Fluorescent compound	Not required	Insoluble
NBT	Nitroblue tetrazolium	10 mg/ml	12,300 (580 nm)	Not required	Insoluble
XTT	2,3-Bis(2-methoxy-4-nitro-5-sulfophenyl)-2H-tetrazolium-5-carboxanilide inner salt	2.5 mg/ml	21,600–23,800 (470–475 nm)	Recommended	Soluble
MTS	MTS(5-(3-carboxymethoxyphenyl)-2(4,5,-dimethyl- thiazolyl)-3-(4 sulfophenyl)tetrazolium inner salt	2.0 mg/ml	26,900 (490 nm)	Recommended	Soluble
WST-1	2-(4-Iodophenyl)-3- (4-nitrophenyl)-5-(2,4-disulfophenyl)-2H-tetrazolium	10 mg/ml	37,000 (438 nm)	Required	Soluble
WST-3	2-(4-Iodophenyl)-3-(2,4-dinitrophenyl)-5-(2,4-disulfophenyl)-2H-tetrazolium	10 mg/ml	30,000 (433 nm)	Required	Soluble
WST-8	2-(2-methoxy-4-nitrophenyl)-3-(4-nitrophenyl)-5-(2,4-disulfophenyl)-2H-tetrazolium	50 mg/ml (H_2_O) 10 mg/ml (DMSO)	30,7000 (460 nm)	Required	Soluble

*Data were compiled from the following references and websites: Sutherland and Learmonth (1997); Berridge et al. (2005); Grela et al. (2018); www.sigmaaldrich.com, www.interchim.com, www.medchemexpress.com, www.abcam.com. DMSO, dimethyl sulfoxide; NA, not applicable.*

Tetrazolium reduction is considered to be proportional not only to the number of cells present but also to their metabolic activity. Indeed, inactive cells (even in large numbers) will have minimal (if any) reduction activity. This can be used to determine the respective proportion of active and inactive cells in various samples by comparison to microscopy count, for example (Dufour and Colon, 1992; Haglund et al., 2002; Bartosch et al., 2003). Similarly, studies have shown that the production of formazan per cell increases following an increase in growth rate (Villegas-Mendoza et al., 2019). On the contrary, at a later stage of batch culture (late stationary phase), where most cells are not growing anymore, staining of such cells by tetrazolium was poor (Fukui and Takii, 1989). Also, it must be noted that different tetrazolium salts might be reduced differently, making comparisons between studies difficult (Trevors, 1984). This adds to the complexity and needs to be clarified when using, comparing, or reviewing tetrazolium studies data.

In addition, there are other limitations to the use of tetrazolium-based dyes that must be taken into account, especially for environmental studies. Several tetrazolium salts [especially 5-cyano-2,3-di-(*p*-tolyl)tetrazolium chloride (CTC), but also iodonitrotetrazolium chloride (INT) and 2,3- bis(2-methoxy-4-nitro-5-sulfophenyl)-2h-tetrazolium-5-carboxanilide inner salt (XTT); Table 1] have been shown to be toxic to some bacteria (Ullrich et al., 1996; Servais et al., 2001; Hatzinger et al., 2003; Nielsen et al., 2003b; Villegas-Mendoza et al., 2015). Similarly, some viable and culturable bacteria may not incorporate formazan derived from CTC or INT, even after long incubation times, thus emphasizing that some bacterial strains lack the ability to reduce tetrazolium to formazan. In environmental studies, it was shown that in some samples, unlabeled (unstained) bacteria could still be responsible for a large fraction of the metabolic activity observed (Servais et al., 2001). Therefore, in order to improve the results, the staining kinetics and tetrazolium concentration must be optimized for different environments and cell types (Servais et al., 2001). Also, this implies that tetrazolium reduction for the estimation of environmental sample microbial activity is indicative of the most active microbial populations, whereas the contribution of fungal and other eukaryotic microbes to the measurement of dehydrogenase activity is extremely limited (Kumar and Tarafdar, 2003).

Finally, depending on the studies considered, reduction of tetrazolium dyes is reported either as rates of reduction (i.e., moles of formazan produced per unit of time, and eventually per mass or volume of sample) or as amount of formazan released [i.e., optical density (OD) or moles of formazan]. Again, it is important to compare rates with other matching rates, for example, formazan production rates with oxygen consumption rates (see example in Martínez-García et al., 2009; Maldonado et al., 2012). Similarly, amounts such as cell number (plate or microscopy count) should be compared to the amount of formazan produced (see example in Oren, 1987).

### Using Tetrazolium Derivatives to Assess NAD^+^, NADH, NADP^+^, and NADPH

**FIGURE 2:**
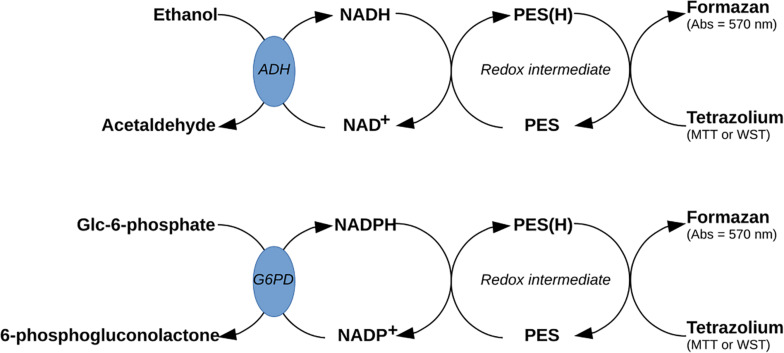
Possible use of tetrazolium assay to assess the metabolic state of cells using ratios of nicotinamide adenine dinucleotides (NAD^+^-NADH) or their phosphorylated derivatives (NADP^+^-NADPH). ADH, alcohol dehydrogenase; G6PD, glucose-6-phosphate dehydrogenase.

Nicotinamide adenine dinucleotides (NAD^+^/NADH) and their phosphorylated derivatives (NADP^+^/NADPH) are important redox-active molecules used in many catabolic and anabolic reactions. Ratios of NAD^+^ to NADH and NADP^+^ to NADPH are therefore considered key indicators of the overall intracellular redox potential and metabolic state. Because tetrazolium salts are often used to assess the activity of NADH or NADPH utilizing enzymes (Figure 1), it is also possible to assess the amount of NAD^+^, NADH, NADP^+^, and NADPH using the redox nature of tetrazolium salts coupled with an appropriate enzymatic system (substrate and enzyme) and electron mediator (redox intermediate) (Figure 2) (Kern et al., 2014; Spaans et al., 2015; Veskoukis et al., 2018). The assay is usually performed on tissue or microbial pellet extracts.

For NADP^+^ and NADPH, the assays rely on the conversion of glucose-6-phosphate (G6-P) to 6-phosphogluconolactone by glucose-6-phosphate dehydrogenase (G6PD; EC: 1.1.1.49), where the simultaneous reduction of NADP^+^ to NADPH is coupled to the reduction of a tetrazolium salt [often thiazolyl blue tetrazolium bromide (MTT) or tetrazolium-based dye (WST); Table 1] through a redox intermediate [phenazine ethosulfate (PES)] (Figure 2). Similarly, for the determination of NAD^+^ and NADH, the assays use ethanol dehydrogenase (ADH; EC: 1.1.1.1), which catalyzes the conversion of ethanol to acetaldehyde. In this reaction, the reduction of NAD^+^ to NADH is also linked to the reduction of a tetrazolium salt to its formazan derivative through PES (Figure 2) (Wagner and Scott, 1994; Kern et al., 2014).

Oxidized (NAD^+^, NADP^+^) and reduced (NADH, NADPH) forms can be assessed separately, as the oxidized forms are easily destroyed by heating at 60°C for 30 min. Alternatively, alkaline (NaOH 0.2 M) or acidic (HCl 0.2 M) treatment in combination with milder heating (55°C for 10 min) allows selection of the forms that will be assayed. However, proper neutralization of the added acid or base needs to be performed (Wagner and Scott, 1994; Kern et al., 2014).

The assay is convenient and easy to perform (especially when using a commercial kit). However, due to the reactivity of the NAD^+^ and NADP^+^ extracts, it is recommended to store them at −80°C after extraction for a maximum of 1 week, or they can be stored on ice for 1 h. Similarly, for complex (food or environmental) samples, attention must be paid to the presence of inhibitory compounds that might alter the results of the enzymatic assay. Furthermore, the complete buffer should be prepared fresh for each measurement. However, commercial assays have procedures that simplify the preparation steps and make the assay very reproducible under ideal conditions (see assay leaflet from producer’s website). Still comparing samples spiked with NAD^+^, NADP^+^, NADH, or NADPH with unspiked samples might allow determining whether a commercial kit is usable under specific conditions, in particular for complex samples.

### Resazurin (alamarBlue^TM^)

**FIGURE 3:**
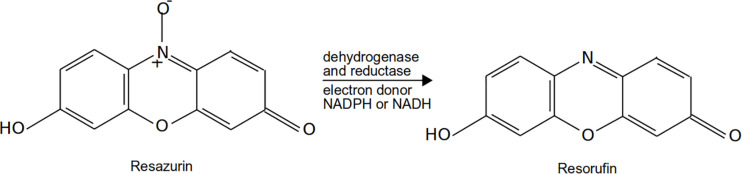
Example of reduction of resazurin (alamarBlue) into resorufin.

Resazurin (7-hydroxy-3H-phenoxazin-3-one 10-oxide) has been used in the composition of many media for anaerobes because of its blue-violet color to indicate the presence of oxygen, which transitions to colorless when no dissolved oxygen is available. Resazurin is also used for metabolic and viability assays (Liu, 1983; Lall et al., 2013; Heller and Edelblute, 2018). Similar to tetrazolium salts, the dye is reduced by NAD(P)H-dependent oxidoreductases and dehydrogenases (Figure 3). Due to the chromogenic and fluorogenic properties of its oxidized form (resazurin) and reduced form (resorufin: 7-hydroxy-3H-phenoxazin-3-one), resazurin can be used for both types of assay. Assays can be performed colorimetrically, as the resazurin changes from blue to violet (molar absorption coefficient of resorufin = 54,000–58,500 cm^–1^⋅M^–1^). Similarly, resazurin is only weakly fluorescent as its irreversible reduction to resorufin makes it highly fluorescent, thus allowing very sensitive detection with a fluorometer. For this purpose, resazurin has been sold in assay kits under different trademarks, such as alamarBlue, PrestoBlue, and UptiBlue, among others. However, it must be noted that such commercial kits are only tested on standard microorganisms (such as *Escherichia coli, Bacillus subtilis, Staphylococcus aureus, Candida albicans*) and standard mammalian cell lines. Therefore, their application to the study of nonstandard microorganisms and environmental samples must be validated/calibrated by the experimenters before use (see examples below).

For environmental conditions and with pure culture, resazurin reduction has been shown to be highly correlated to oxygen consumption (Liu, 1983; McNicholl et al., 2007; González-Pinzón et al., 2012). Indeed, resazurin assays work best for aerobic or microaerophilic microorganisms. For example, *Clostridium butyricum* (a strict anaerobe) does not reduce resazurin (Karakashev et al., 2003) like other strictly anaerobic *Clostridium* are reported to reduce tetrazolium salts (List et al., 2019), maybe through other processes. Furthermore, we can safely assume that resazurin is not likely to be reduced by anaerobes (or during anaerobic respiration, such as sulfate or nitrate reduction), as it is used in media as an oxygen redox indicator. This makes the application of resazurin for assays a bit more limited compared to tetrazolium salts. Still, when looking at an aerobic (or microaerobic) setup, resazurin is a very sensitive assay, especially when used with fluorometry. Another limitation to the use of resazurin is its toxicity toward certain cells. Indeed, the toxicity is likely to be low for most microbes as the concentration of resazurin in the assay (0.4–4 mM) is lower than the concentrations at which inhibition is observed for some bacteria (Schmitt et al., 2013). Still, some studies have mentioned its toxicity toward bacteria present in raw milk, pathogens, and human cell lines, especially tumor cell lines (Ramsdell et al., 1935; Pace and Burg, 2013; Schmitt et al., 2013). Finally, the presence of nanomaterials has been shown to influence resazurin reduction and its fluorescence, and much care has to be taken in the presence of such materials (Breznan et al., 2015).

## Adenosine Triphosphate Assays

Adenosine triphosphate (ATP) is a crucial molecule in microorganisms because of its role as a universal energy currency. However, it must be noted that ATP on its own can only reveal a tiny fraction of the energetic state of a cell or a system as other adenylates [adenosine diphosphate (ADP) and adenosine monophosphate (AMP)] have to be accounted for (see below). When considering only ATP, the general assumption is that microorganisms generate ATP through catabolic reactions and subsequently use it for “housekeeping,” growth, and replication. As a result, ATP is believed to indicate the presence of viable microorganisms in samples. Alternatively, as the intracellular concentration of ATP per cell has been shown to vary with changing environmental and physiological conditions, it can be used as a proxy to indicate the metabolic activity of cells (Schneider and Gourse, 2004; Mempin et al., 2013; Nescerecka et al., 2016). Indeed, in cells losing viability, the ability to synthetize ATP is lost and many biochemical reactions including the action of ATPases rapidly deplete any remaining ATP from the cytoplasm. In growing (viable) *E. coli* and *Salmonella typhimurium*, the intracellular concentration of ATP varies from 1 to 5 mM (Mempin et al., 2013). For a wider range of bacteria, values are between 0.1 and 26 fg ATP⋅CFU^–1^ (Kodaka et al., 1996; Venkateswaran et al., 2003); however, the average is usually around 1 fg ATP⋅CFU^–1^. The values can be converted to mM by assuming a cytoplasm volume^1^ of 0.67 μm^3^; however, one has to note that cell volume varies a lot between microbial species and within the same species with varying growth conditions. The values for *Bacillus* species spores are much lower, ranging from 0.01 to 0.0002 fg ATP⋅CFU^–1^; however, in *Bacillus* spores, rather high levels of 3-phosphoglyceric acid (3PGA), a potential rapid source of ATP, are found (Ghosh et al., 2015; Setlow and Johnson, 2019). Finally, for the yeast *C. albicans*, it is much higher, 213 fg ATP⋅CFU^–1^. Of note, recent progress in single-cell imaging has shown that within the same culture, there was a fair amount of variation (from less than 0.5 to 14 mM) in the distribution of ATP concentration within individual cells (Yaginuma et al., 2014). Furthermore, the release of extracellular ATP has to be taken into account when assessing metabolism or viability, as a large percentage of the ATP in a culture can be extracellular, especially in the exponential growth phase or when exposed to disinfectants such as chlorine (Mempin et al., 2013; Nescerecka et al., 2016). Therefore, ATP values have to be taken with care, especially as their use is still debated for some applications. Indeed, the interpretation of ATP measurements, especially with respect to viability and activity, can be improved if the other adenylates (ADP and AMP) are taken into account (or other parameters such as biomass or cell count). The concept of adenylate energy charge (AEC) was introduced by Atkinson (1969) to reflect the fact that metabolic processes are sensitive to levels of individual adenine nucleotides or their relative proportions. AEC is defined as [(ATP) + 0.5 (ADP)]/[(ATP) + (ADP) + (AMP)]. However, in some studies, it is simplified as (ATP)/[(ATP) + (ADP)]; thus, it is worth checking which one is used for comparison purposes. Still, it is often considered that the ratio of ATP, ADP, and AMP is functionally more relevant than the absolute concentration of ATP alone (De La Fuente et al., 2014). With respect to the value of AEC, it is generally considered that metabolically active and/or growing cells have AEC values between 0.70 and 0.95 (Lee and Colston, 1986; De La Fuente et al., 2014). Similarly, cells remain viable with AEC values of 0.5–0.8. Finally, in stress conditions, AEC falls below 0.5, and values below 0.5 are usually considered to be incompatible with maintaining the minimal level of homeostasis required for viability (Chapman et al., 1971). Still, *Bacillus* endospores can have an AEC value of 0.08, for example (Kodaka et al., 1996). AEC has been subjected to intense discussions and is considered as “misleadingly simplistic.” Still, many authors agree that it can be a valuable approach to assess the broad homeostatic feature of ATP-utilizing and ATP-replenishing reactions (Purich and Allison, 1999).

**FIGURE 4:**
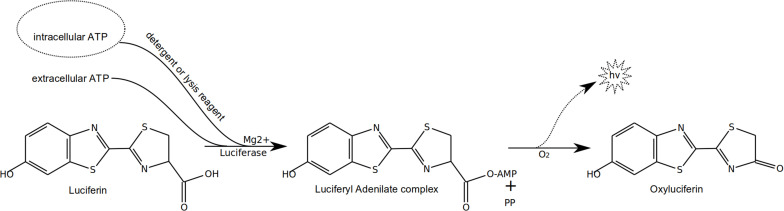
Simplified reactions occurring during ATP assay using firefly luciferase.

Measurement of ATP can be performed using two types of commercial enzymatic assays. The first ones rely on the use of luciferase (firefly luciferase EC 1.13.12.7 or other luciferase) to oxidize luciferin into oxyluciferin with concomitant use of ATP and production of AMP. The overall reaction (Figure 4) releases light that can be measured by a luminometer. Many assay kit reagents contain a detergent to allow cell lysis and recovery of intracellular ATP, as well as ATPase inhibitors to avoid loss of ATP during the processing. Still, for very “dirty” or some environmental samples, care has to be taken that impurities do not contain other compounds that might also affect the level of ATP extracted and/or measured (see below as well). This assay is by far the most popular ATP assay used. At the end of this assay, remaining adenylates (ADP and AMP) can be quantified by converting them enzymatically into ATP using pyruvate kinase (EC 2.7.1.40 for ADP) or pyruvate kinase and adenylate kinase (also called myokinase; EC 2.7.4.3 for AMP) (Lee and Colston, 1986; Kinniment and Wimpenny, 1992) and performing an additional luminescence measurement. The values can be used to calculate AEC (see above). Commercial kits are available for determination of ATP only or sequential determination of all three adenylates.

**FIGURE 5:**
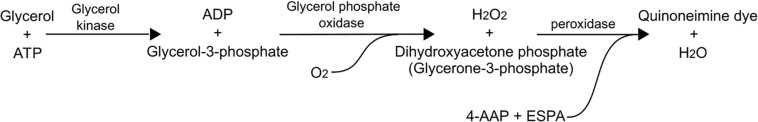
Possible use of glycerol phosphorylation to assess ATP.

Colorimetric alternatives are also available. Among the alternatives, glycerol phosphorylation reaction can be used to assess the amount of ATP present. Glycerol added in excess is phosphorylated by glycerol kinase (EC: 2.7.1.30) to produce glycerol-3-phosphate and ADP. The glycerol-3-phosphate is then further oxidized by glycerol phosphate oxidase (EC: 1.1.3.21), producing dihydroxyacetone phosphate (glycerone phosphate) and hydrogen peroxide (H_2_O_2_). Finally, a peroxidase (EC: 1.11.1.7) catalyzes the redox-coupled reaction of H_2_O_2_ with 4-aminoantipyrine (4-AAP) and *N*-ethyl-*N*-(3-sulfopropyl)-*m*-anisidine (ESPA), leading to the formation of a purple dye that is measured at 540 or 570 nm (Figure 5). Again, correct extraction of ATP and use of detergent are important to recover ATP.

The advantage of the ATP assay is that you do not have to rely on an incubation step with a population of viable cells to convert a substrate (such as tetrazolium, resazurin, or FDA) into a colored compound. In addition, for some applications (mostly with cultures), there is also no need to remove cell culture medium or wash cells before adding the reagent, which can be fully automated for high throughput. Still, serial measurements are needed to assess the flow of intracellular and extracellular ATP and get a complete image of metabolic processes over time. If sample processing needs to be performed at a later stage (i.e., after all samples have been collected), it is recommended to snap-freeze the samples in liquid nitrogen or on dry ice. For this assay, ATP extraction remains a crucial step and different extraction methods were tested on microbial culture samples, showing strong variation in ATP recovery (Lundin and Thore, 1975; Prioli et al., 1985; Stanley, 1989). Still, a comparison between commercial luciferase-based ATP assays and 31P NMR ATP determination (which does not require an extraction step) on blood cells did not show significant differences (Marjanovic et al., 1993). For environmental samples, however, the determination of ATP is more difficult due to the poor recovery rate of some extraction methods (Karl and Holm-Hansen, 1978; Webster et al., 1984) and potential hydrolysis or other chemical interactions (Karl and Holm-Hansen, 1978; Karl, 1993). In addition, early studies on marine samples using charcoal columns found that it is likely that part of the ATP is hydrolyzed during extraction and recovery. It is also mentioned that filtration-induced metabolic stress might lead to a decrease in ATP (however, total adenylate content remains stable). The exact process leading to ATP hydrolysis remains unclear; still, much care has to be taken when analyzing environmental samples and drawing conclusions about activity (or biomass). Also, for all types of samples, the pH must be controlled with care, as it has been shown to affect the results (Posimo et al., 2014; Šimèíková and Heneberg, 2019).

## Fluorescein Diacetate and Derivative Compounds Hydrolysis

**FIGURE 6:**
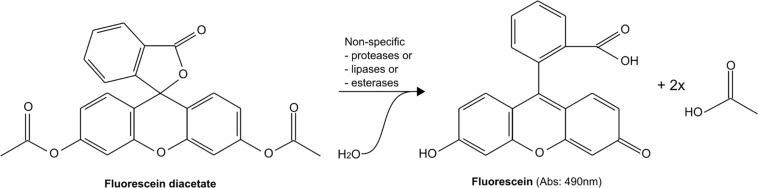
Example of hydrolysis of fluorescein diacetate to form fluorescein.

Fluorescein diacetate (3′,6′-diacetyl-fluorescein) hydrolysis is considered to be a simple and affordable method of estimating microbial activity in various samples, including soils, sludges, marine sediments, and cell cultures (Fontvieille et al., 1992; Yuan et al., 2007; Sánchez-Monedero et al., 2008; Gómez et al., 2015; Mahu et al., 2018). In this assay, FDA, which is colorless, is hydrolyzed by nonspecific esterases, proteases, and lipases into green-colored fluorescein (Figure 6). The enzyme performing such hydrolysis can be free or membrane-bound (Fontvieille et al., 1992; Adam and Duncan, 2001). As with tetrazolium, there are several FDA-based compounds, such as 5(6)-carboxyfluorescein diacetate (carboxy-FDA) and 2′,7′-dichlorofluorescein diacetate (chloromethyl-FDA), leading to the formation of different hydrolyzed products, in this case, 5-carboxyfluorescein (carboxyfluorescein) and 2′,7′-dichlorofluorescein (chloromethyl-fluorescein). However, unlike tetrazolium salt or resazurin reduction, FDA hydrolysis is not expected to be directly linked to O_2_ consumption, although many studies have shown that the two are often correlated (Fontvieille et al., 1992). Still, the assay can be widely used in many setups as FDA hydrolysis capacity is widespread (Schnurer and Rosswall, 1982; Gaspar et al., 2001; Prosser et al., 2011; Liang et al., 2019; Long et al., 2019; Braun et al., 2020). Intracellular hydrolysis of FDA results in the accumulation of fluorescein (which is unable to pass cell membranes) in metabolically active cells. As a consequence, FDA hydrolysis has often been used in combination with ethidium bromide or propidium iodide penetration in damaged cells and binding to DNA in microscopic assays of viability (see review in Tawakoli et al., 2013). Such microscopic assays are often referred to as LIVE/DEAD staining, although LIVE/DEAD is a trademark of Invitrogen, Thermo Fisher Scientific. LIVE/DEAD staining has been benchmarked only for proteobacterial pathogen (mostly *E. coli*) and has been reported to be prone to artifacts, including incomplete stain penetration or false staining of live cells as dead (Stewart and Franklin, 2008) in many cases. Similarly, FDA hydrolysis and its accumulation in active cells is often used in combination with fluorescence-activated cell sorting (FACS) or fluorescence microscopy to assess the number of stained cells and their relative fluorescence (Hoefel et al., 2003). FACS and LIVE/DEAD assays will not be further discussed here, and focus will remain on bulk assays.

To measure metabolic rates in environmental samples or, more rarely, in cultures, FDA hydrolysis is initiated by adding the FDA in a buffer (usually 60 mM phosphate buffer) to the sample. After a determined incubation period, the reaction is stopped using rather large amounts of organic solvents [chloroform:methanol (2:1) or acetone]. In many environmental samples, the solvent mixture also serves as a fluorescein extractant by dissolving membranes (Adam and Duncan, 2001; Gaspar et al., 2001; Patle et al., 2018). In cell cultures, the same methodology can be applied, or Triton X-100 can be used to permeabilize yeast cells, for example (Breeuwer et al., 1995). After extraction, the fluorescein amount is quantified by measuring either fluorescence or absorbance. Although FDA and its derivatives are mostly used for viability assays, their use as proxies to measure microbial metabolism remains very interesting, as the molar absorption coefficient of fluorescein dye (E_490_ nm) lies between 67,000 and 79,000 cm^–1^⋅M^–1^ (Mota et al., 1991). Such a high absorption coefficient compared to formazan (see above and Table 1) and its lower toxicity make it interesting for environmental studies. In addition, fluorometric detection is even more sensitive and can be used as well. However, the good detection of fluorescein is slightly compensated for by the lower water solubility of FDA. Indeed, many protocols recommend diluting FDA in an organic solvent such as acetone, chloroform, dimethyl sulfoxide (DMSO), ethanol, or methanol where the solubility is about 25 mg/ml.

## Electron Acceptor Consumption Rate

Electron acceptors are key players in microbial metabolism, providing energy for many other processes. Indeed, many microorganisms obtain their energy from redox reactions. Among these redox reactions, aerobic respiration, anaerobic respiration, and oxidation of reduced inorganic compounds rely on redox couples such as O_2_/H_2_O, NO_3_^–^/NO_2_^–^ and Fe^3+^/Fe^2+^, for example (Konhauser, 2007; Oren, 2008). The consumption rate of the electron acceptor or the production rate of its reduced form is therefore a very good proxy to follow or estimate microbial metabolism. As a result, many assays and techniques have been used for this purpose. Again, many of these assays were intended for use with pure culture, and their use for environmental samples should be carefully evaluated, as there could be many limitations or artifacts. The next sections will provide insights on some of the commonly used assays; however, such a list cannot be considered as exhaustive. In particular, note that the focus is put on terminal electron acceptors and that NAD(P)^+^/NAD(P)H have been discussed previously in the text.

### Oxygen

Oxygen is among the most used proxies to assess the metabolic activity of various organisms. This is also true for microorganisms and microbial oxygen consumption rates, and their measurement could be the topic of a review by itself. As a result, many methods have been developed to assess oxygen consumption by heterotrophs or production by photosynthetic microbes (see review in Renger and Hanssum, 2009). Indeed, oxygen concentration can be measured in solution using electrodes (electrochemical sensors), optodes (optical-based sensors), or chemical assays such as the Winkler titration or directly in the headspace of sealed vials using laser spectrometry (Bondyale-Juez et al., 2017).

With respect to dissolved oxygen, the Winkler titration (Winkler, 1888; Katznelson, 2004) was considered to be the most accurate method for a long time (Bittig et al., 2018); however, the method is demanding and rather time-consuming, as the reaction of dissolved oxygen with manganese is rather slow and can take up to 30 min. Therefore, the Winkler titration is difficult to apply serially to assess the rapid consumption of oxygen. As a result, optical and electrochemical sensors have been preferred in many cases (Kemker, 2014; Bondyale-Juez et al., 2017), especially as some systems have been introduced as 96-well plates (Guarino et al., 2004; Dike et al., 2005). Nowadays, both optical- and electrochemical-based sensors have very good performance and can sense dissolved oxygen in an aqueous system easily and accurately, usually with an error below 1% (Briand et al., 2004; Tengberg et al., 2006; Renger and Hanssum, 2009). In addition, both types of electrodes can be built as macro or micro types of sensors (Revsbech et al., 1983; Visscher et al., 1991; Glud et al., 1999, 2005). Still, it is worth reviewing the minor differences between these types of sensors. Clark-type electrodes have been shown to require frequent calibration, to consume some oxygen, and to be sensitive to environmental factors (especially salinity, flow, temperature, and pressure). On the other hand, oxygen optodes do not consume oxygen and the signal is minimally affected by flow velocity and other environmental factors, except temperature. However, in many commercial optodes, the temperature dependence is automatically corrected by an attached thermistor^2^. In the end, the most important difference between optodes and electrode is the possibility to obtain 2D spatially resolved information by building planar optodes (Glud et al., 2005; Staal et al., 2011; Tschiersch et al., 2011; Farhat et al., 2015), as microelectrodes (including needle optodes) are limited to 1D depth profiles or time series (Kuhl, 2005; Cotter et al., 2009; Riedel et al., 2013). Finally, a few commercial high-throughput products have been released using optodes (or optode-like technology) and allowing 96- or 384-well-plate format assays. Among them, the BD Oxygen Biosensor System is an oxygen-sensing microplate using silicone and an embedded fluorophore [tris 4,7-diphenyl-1,10-phenanthroline ruthenium (II) chloride] at the bottom of the wells. The instrument has been used successfully to monitor the growth and oxygen uptake rates of mammalian cell cultures and bacterial cultures (Stitt et al., 2002; Guarino et al., 2004). A similar system, the Agilent Seahorse XF Analyzer, offers more analytical capacities but is intended for work with adherent cells (Gerencser et al., 2009), and as a result, very little data have been obtained using this system on bacteria. Still, parasite investigations have been performed successfully (Shah-Simpson et al., 2016; Gonzalez-Ortiz et al., 2019).

Finally, although the measurement is a bit more indirect compared to dissolved oxygen measurement, oxygen can also be assessed in the headspace of sealed gas-tight vials using laser spectroscopy. Indeed, tunable diode laser absorption spectroscopy (TDLAS) can be used to measure several gases in gas phase. TDLAS measures the absorption of laser light in the near- to mid-infrared wavelength. Detection of gases can be improved using techniques such as wavelength modulation spectroscopy (WMS) or frequency modulation spectroscopy (FMS) (Krishna and O’Byrne, 2016). It works well for many gases, including oxygen and carbon dioxide. TDLAS readings are fast; however, current instruments have only one measuring channel and samples need to be read over a time series manually. Still, some semiautomated systems are currently available for industrial applications, especially in the pharmaceutical industry (Brückner et al., 2019). In addition, such instruments are under a constant flow of nitrogen and therefore require a fair amount of that gas. TDLAS has been used in microbiology recently for the detection of bacterial growth in pharmacological samples and clinical samples (such as blood cultures) based on O_2_ and CO_2_ concentrations in vials (Brueckner et al., 2016, 2017; Duncan et al., 2016; Shao et al., 2016, 2018). Furthermore, it is possible to detect bacterial growth linked to cystic fibrosis or the presence of *Helicobacter pylori* infection directly from exhaled air (Henderson et al., 2018) using other volatile compounds.

### Nitrates (No_3_^–^) and Nitrites (No_2_^–^)

**FIGURE 7:**
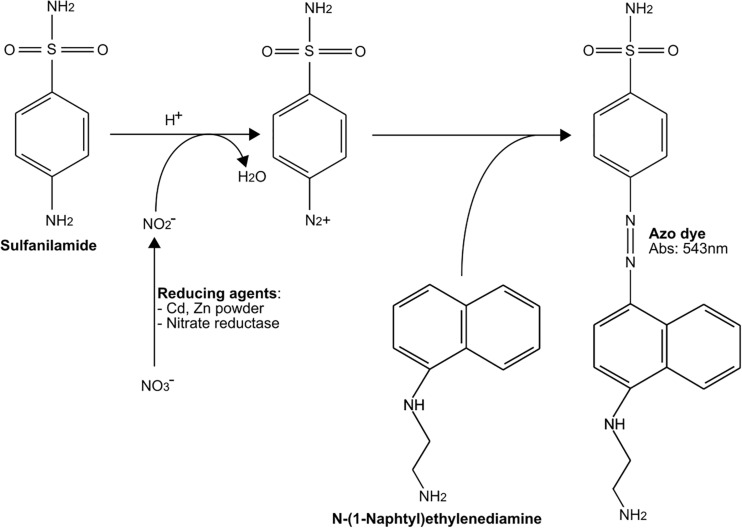
Scheme showing reactions involved in determination of nitrite and nitrate. Some reducing agents to convert nitrate to nitrite are listed (see text for a more complete list).

Because the energy yield of nitrate reduction is still rather high compared to other types of anaerobic metabolism, it is a commonly encountered type of metabolism that is also found in pathogenic microbes. As a result, nitrate reduction is often studied in microbiology and assessed in several ways. Although nitrate (NO_3_^–^) and nitrite (NO_2_^–^) can be measured by high-performance liquid chromatography (HPLC), ion chromatography (IC), or ion-specific electrodes, the Griess reaction (Griess, 1879) remains very popular to assess the potential for nitrate reduction and, if measured serially, denitrification rates. The original Griess reaction assesses the amount of nitrite in the sample in two steps. First, nitrite reacts under acidic conditions with sulfanilic acid, thus producing a diazonium cation. Second, the diazonium cation reacts with 1-naphthylamine to produce a dark red water-soluble azo dye (Tsikas, 2007) (Figure 7). The Griess reaction is specific for nitrite but can determine nitrate as well if it is properly reduced to nitrite chemically or enzymatically. Such reduction is usually performed by adding zinc powder to the sample solution. However, alternative reduction methods using cadmium, vanadium, silver, or nitrate reductase have been considered as well (Sun et al., 2003; Beda and Nedospasov, 2005; García-Robledo et al., 2014; Wang S. et al., 2016).

Like many assays, the Griess reaction has been modified, and the original sulfanilic acid and 1-naphthylamine were replaced by sulfanilamide and *N*-(1-naphthyl)ethylenediamine (NED or NEDA). Most variations of the Griess assay were reviewed in Tsikas (2007).

Finally, it must be noted that the low cost and simplicity of the Griess reaction have attracted the attention of a wide spectrum of microbiologists ranging from soil scientists to clinical microbiologists.

### Sulfate and Sulfide

For sulfate-reducing and sulfide-oxidizing bacteria, sulfates and sulfides can be assessed by using HPLC or IC. In addition to these methods, which are used a lot, there are also simple assays. For sulfate, barium sulfate precipitation is a very simple assay that uses the low solubility of this salt [solubility at 20°C = 2.42⋅10^–3^ g⋅L^–1^ − Ksp = 1.0842⋅10^–10^ (25°C)], which is measured turbidimetrically (Coleman et al., 1972; US EPA, 1986; Kolmert et al., 2000). As a consequence, the precipitation is often carried out using centrifuged or filtered samples. Moreover, to ensure similar precipitation conditions (and uniform crystal size distribution to improve turbidimetric measurement reproducibility) between samples, a conditioning reagent containing sodium chloride, ethanol, glycerol, and hydrochloric acid is used (its composition varies between authors and between available commercial assay kits). Many authors have reported that the assay is rather long and not appropriate for high-throughput measurement. Still, for slow-growing sulfate-reducing bacteria (SRB), the assay allows serial measurements and determination of the sulfate reduction rate.

In the case of bacteria reducing sulfide to elemental sulfur, sulfides are more practical to measure, as many methods have been developed and can be applied to biological samples (see review in Lawrence et al., 2000). In particular, hydrogen sulfide (HS^–^) ion-sensitive electrodes are very practical and are often used because they are available in macro and micro format. With microelectrodes that are used *in situ* (or *ex situ*), measurements are taken directly and possibly on-site. They have been extensively used in sediment, microbial mat, and biofilm studies (Ito et al., 2002; Farías et al., 2014; Pages et al., 2014; Wong et al., 2015). However, for macroelectrodes, especially if measurement is delayed until the return to the lab, transportation and potential aeration might bias the results, as sulfides will oxidize rapidly. To prevent such oxidation, commercial sulfide antioxidant buffer (SAOB) or 10 M NaOH can be used. Solutions such as zinc acetate should be avoided, as ion-selective electrodes only measure free sulfides.

On the other hand, sulfide assays relying on the formation of methylene blue have been very popular in microbiology (Reese et al., 2011). These assays rely on the reaction of dimethyl-paraphenylene diamine salts with sulfide to form methylene blue under acidic conditions. Absorbance of the methylene blue formed can be measured at 663 nm (E_663_ nm = 95,000 cm^–1^⋅M^–1^; Cenens and Schoonheydt, 1988). To prevent oxidation of the sulfide, the samples can be fixed as ZnS using zinc acetate solution. The ZnS formed is usually stable for 1 month. Upon acidification, ZnS can be redissolved and assessed as described above. Many modifications of the assay have been used over time (see review in Lawrence et al., 2000; Reese et al., 2011). However, in microbiology and the study of microbial mats, the modification of Pachmayr in 1960 (Pachmayr, 1960) using dimethyl-paraphenylene diamine sulfate (DPDS) is one of the most commonly used (Trüper and Schlegel, 1964; Gallagher et al., 2012). Although such assay is more work-intensive compared to ion-selective electrode (ISE) readings, the fact that the sample can be fixed and processed at a later time point (up to 1 month) makes it useful in practice, as large batches of samples can be processed at the same time. Furthermore, with respect to environmental studies, the assay is sensitive to metastable forms of iron sulfide deposits [such as amorphous FeS nanoparticles, Mackinawite (FeS), Greigite (Fe_3_S_4_), or nanoparticle of pyrite (FeS_2_)] that might be present on cell surfaces or attached to biofilms/extracellular polymeric substance (EPS) matrix (Morse and Rickard, 2004; Rickard and Morse, 2005). In human microbiology, the assay has also been adapted for sulfate reduction occurring in the oral cavity responsible for oral malodor (Kanehira et al., 2012).

### Metal and Metalloid Ions

Many metal ions can be biologically reduced (and less frequently oxidized), thus releasing sufficient amounts of energy to sustain growth processes. Among those metals, Fe, Mn, V, Cr, Cu, Mo, As, Hg, Se, Au, U, and Tc have been reported to be reduced by microorganisms (Lovley, 1993; Konhauser, 2007; Oren, 2008). Among them, iron, which is the fourth most abundant element in the Earth’s crust, has received special attention, as Fe(II) can function as an electron source and Fe(III) as a terminal electron acceptor under anoxic conditions for iron-reducing microorganisms. In this context, iron redox reactions have the potential to support substantial microbial populations in soil and sedimentary environments. Although ion-selective electrodes have been developed for many of these metals (Gupta et al., 2011), the commercial versions of these electrodes are limited to a few of the metals (Cu, Hg, Ag, Cd). Therefore, atomic absorption or MS is often used to measure their concentration. As these methods are not always available in a microbiology lab and because some of the samples cannot be stored for a long period, reduction rates are rarely measured with this type of metabolism. Still, nonspecific assays (such as those described above, FDA, or calorimetry; see below) can be useful in such contexts.

Only iron (i.e., Fe^2+^ and Fe^3+^) can be assessed easily using assays relying on the reaction of a chromogene with iron II (phenanthroline, bathophenanthroline, ferrozine, and ferrene) (Saywell and Cunningham, 1937; Goodwin and Murphy, 1966; Hirayama and Nagasawa, 2017; Hopwood et al., 2017; Hach, 2020). Many of these assays are available as commercial kits and often allow the measurement of Fe^2+^ and total Fe by adding a reducing agent (ascorbic acid or ammonium hydroxide) to convert Fe^3+^ into Fe^2+^. Many commercial kits do not provide the composition or the type of assay present in the kit. However, it is possible to decipher them according to the measurement wavelength of the assay (phenothroline and bathophenanthroline: 533–535 nm; ferrozine: 563 nm; ferrene: 593 nm; Hirayama and Nagasawa, 2017). In some assays, this can even be performed serially, making the assays very convenient. Indeed, some commercial kits, such as the HACH test kit (LCK 320), have all the reagents contained in one single assay tube with breakable septa. In other assays, the determination of Fe^2+^ and total Fe must be performed in parallel. The uses, advantages, and drawbacks of these assays have been reviewed by a geomicrobiologist (Braunschweig et al., 2012). Usually, organisms that grow using iron redox reactions are rather slow, and these assays are rapid and practical enough to be performed serially to determine iron oxidation or reduction rates.

## Carbon Source Consumption and By-Product Release Rates

### Carbon Sources

Carbon sources are the key to microbial metabolism, providing carbon for biosynthetic processes but also for energy metabolism in many cases. Current technologies (such as Biolog EcoPlate) using well-plate readers allow rapid screening of several important carbon sources for their utilization and eventually link such data with metagenomics data obtained separately (Lyons and Dobbs, 2012; Uroz et al., 2013; Gryta et al., 2014; Feigl et al., 2017). However, such an approach does not provide information on metabolic rates. Indeed, the rate of carbon source consumption is directly linked to metabolic activity. In this context, it must be noted that metabolic activity is not always related to growth, as microbes are known to engage in futile cycles for several reasons (Neijssel et al., 1990; Russell and Cook, 1995; Qian and Beard, 2006). Therefore, it is important to avoid making direct links between growth, metabolic activity, and potential carbon source utilization. One has to discriminate clearly between metabolic fingerprinting and metabolism measurement. Still, a screening of the important carbon sources using EcoPlates might allow deciphering which assays must be performed at later stages.

The variety of carbon sources that can be used by microbes is indeed matched by a high number of assays to assess them. Especially, there are many enzymatic assays matching the substrates to be assessed. There are also plenty of chemical assays that are useful for assessing the uses of various carbon sources.

### Carbohydrates

**FIGURE 8:**
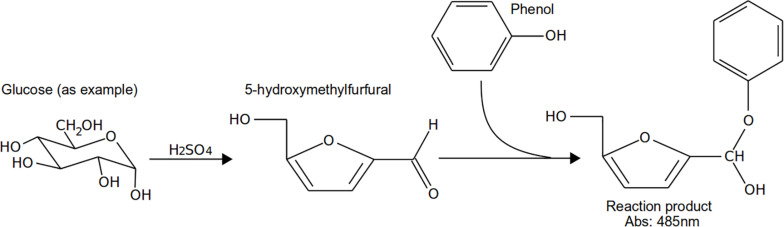
Example reaction of phenol–sulfuric acid assay with glucose.

Carbohydrates represent a large fraction of carbon sources investigated in clinical or natural environments and added to solid and liquid culture media. As a consequence, many chemical assays have been developed to assess the amount of carbohydrates in such samples. All of these assays rely on the same general principle: carbohydrates (including bound carbohydrate and glycoprotein) are hydrolyzed using a strong acid and heat. The reaction mixture contains a developing reagent (phenols, orcinol, *o*-toluidine, anthrone, carbazole) that allows the development of a color measurable by spectrophotomeric means (Georges, 1971; de Toledo et al., 2012). Among these assays, the phenol–sulfuric acid assay from Dubois et al. (1951, 1956) (Figure 8) and the anthrone assay (Dreywood, 1946; Morse, 1947; Trevelyan et al., 1952) have been the most popular for years and are used extensively in many labs. Both assays are inexpensive and work well for monosaccharides, polysaccharides, and complex polysaccharides such as EPS (Scott and Melvin, 1953; Dubois et al., 1956; Braissant et al., 2009). However, the anthrone assay works better for solutions containing a single type of hexose because even sugars with similar structures result in different rates and quantities of color development (Cui and Brummer, 2005). With respect to throughput, the original phenol–sulfuric acid assay is difficult to adapt to microplate format (due to the high temperature reached during the assay, incompatible with polystyrene well plates); however, both that assay and the anthrone assay have been modified extensively and optimized for well plates (Laurentin and Edwards, 2003; Leyva et al., 2008). Often, those modified methods involve some incubation at higher temperature (90°C or above) to speed up the reaction. Many commercial versions of such chemical assays are sold as “carbohydrate assay” or “total carbohydrate assay,” making them readily available. For these assays, samples can be collected serially and frozen. At the end of the experimental measurement, the assay can be performed easily and the well plate format allows rather high throughput. Therefore, carbohydrate consumption rates can be easily determined during an *in vitro* assay where an excess of one sugar is used in the medium.

### Glucose

**FIGURE 9:**
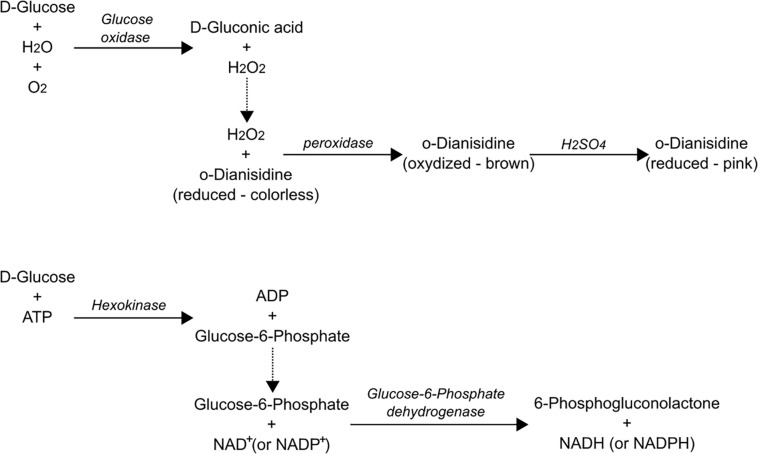
Example of enzymatic assay for glucose using glucose oxidase (top panel) or hexokinase (bottom panel). Note that glucose-6-phosphate dehydrogenase usually uses NADP^+^ as cofactor; however, this enzyme is rather unspecific with regard to the cofactor (NADP^+^ or NAD^+^). Thus, in many commercial assays, NAD^+^ is used because of its lower cost and better stability (Bondar and Mead, 1974; Fuentealba et al., 2016).

Among carbohydrates, glucose is probably the most used and monitored, and it can be easily assessed using the assays described above. However, for samples containing other sugars or interfering substances (toluene, for example; Devor et al., 1964), enzymatic assays are very specific and provide more accurate measurements. Two enzymatic assays exist to assess glucose in various samples (Figure 9). The first assay is based on glucose oxidation by glucose oxidase (EC: 1.1.3.4), which generates H_2_O_2_ and D-gluconic acid. After this step, hydrogen peroxide reacts with *o*-dianisidine in the presence of peroxidase (EC: 1.11.1.7) to form a colored compound in the reaction solution. Finally, oxidized *o*-dianisidine reacts with sulfuric acid to form a more stable colored product (Sigma Aldrich). The other enzymatic assay used for glucose determination relies on hexokinase (EC: 2.7.1.1). Glucose is phosphorylated by hexokinase, and the resulting glucose-6-phosphate is then oxidized to 6-phospho-gluconolactone by glucose-6-phosphate dehydrogenase (EC: 1.1.1.49). The NADH produced results in increased absorbance at 340 nm. Of note, glucose-6-phosphate dehydrogenase usually uses NADP^+^ as a cofactor, but this enzyme is rather unspecific with regard to the cofactor (NADP^+^ or NAD^+^). Thus, in many commercial assays, NAD+ is used because of its lower cost and better stability (Bondar and Mead, 1974; Fuentealba et al., 2016). Both enzymatic assays are commercially available and cost roughly the same. Several studies focused on glucose consumption used diabetic glucose meters [electrochemical sensors also based on glucose oxidase (sensing H_2_O_2_)] to measure glucose. The approach is cheap, although calibration is required as those meters have been optimized for blood tests and might have an offset in other media or solutions (Flavigny, 2014). Glucose-doped samples might also be of use for low-glucose concentrations or when validation is needed (authors’ personal observations). At this point, many attempts are made to “hack” such meters and use them as bacterial contamination detection tools/platforms (Flavigny, 2014; Wang et al., 2015). Finally, it must be noted that glucose sensors are among the most investigated types of sensors. A recent study showed that over 9,000 publications existed on the topic in Web of Science, and around 400 more appear every year (Oliver et al., 2009; Chen et al., 2013). As a consequence, glucose detection will still change quite a lot in the coming years and other assays are likely to become available.

### Organic Acids

Among the potential carbon sources used by microbes or the by-products released by their metabolism, organic acids have received a lot of attention due to their role in food production, plant growth, diseases, and weathering processes. The measurement of organic acids in various samples is often performed using IC or HPLC (Takao, 1965; Adams et al., 1984; Trifirò et al., 1997; Lefebvre et al., 2002; Tsangalis and Shah, 2004; Klinke et al., 2009; Wojtczak et al., 2010) as well as capillary electrophoresis (Kudrjashova et al., 2004). However, chromatography and electrophoresis often demand some preparation, during which sample recovery might not be optimal. For single organic acids, many commercial enzymatic assays can be used to detect the most commonly encountered ones (Table 2). These assays often rely on the production or consumption of NADH directly detected by a change in absorbance at 340 nm. Alternatively, in enzyme assays using dehydrogenases or coupled to peroxidase (EC: 1.11.1.7), it is common to add a redox indicator [such as 2-(4-iodophenyl)-3-(4-nitrophenyl)-5-(2,4-disulfophenyl)-2H-tetrazolium (WST-1) or resazurin; see previous sections] to perform colorimetric/spectrophotometric measurement in the visible range. Many of these assays can be performed at later stages in well plate format, making throughput rather high.

**TABLE 2:** Non-exhaustive list of organic acid assays and their enzymatic and detection systems.

Analyte	Enzymes system used	Measurements	Detection limit
Acetic acid	Acetyl-coenzyme A synthetase Citrate synthase L-Malate dehydrogenase	Formation of NADH Measured at 340 nm	0.14 mg/L (2.3 μM)
Acetic acid	Acetate kinase Phosphotransacetylase Pyruvate kinase D-Lactate dehydrogenase	Consumption of NADH Measured at 340 nm	0.254 mg/L (4.2 μM)
Citric acid	Citrate lyase L-Malate dehydrogenase D-Lactate dehydrogenase	Consumption of NADH Measured at 340 nm	0.491 mg/L (2.6 μM)
Citric acid	Citrate lyase Oxaloacetate decarboxylase D-Lactate dehydrogenase	Formation of NADH NADH reduces redox probe Abs measured at 570 nm (AB)	0.002 mg/L (0.01 μM)
Formic acid	Formate dehydrogenase	Formation of NADH Measured at 340 nm	0.0932 mg/L (0.2 μM)
D-gluconic	Gluconate kinase 6-Phosphogluconate dehydrogenase	Formation of NADH Measured at 340 nm	0.792 mg/L (4.0 μM)
D-Lactic acid	D-Lactate dehydrogenase D-Glutamate-pyruvate transaminase	Formation of NADH Measured at 340 nm	0.214 mg/L (2.4 μM)
D-Lactic acid	D-Lactate dehydrogenase	Formation of NADH NADH reduces redox probe Abs measured at 450 nm (WST)	1.8 mg/L (20 μM)
D/L-Lactic acid	L-Lactate dehydrogenase D-Lactate dehydrogenase D-Glutamate-pyruvate transaminase	Formation of NADH Measured at 340 nm	0.214 mg/L (2.4 μM)
D/L-Lactic acid	Lactate oxidase	Formation of H_2_O_2_ Fluorometric H_2_O_2_ probe	0.135 mg/L (1.5 μM)
D-Malic acid	D-Malate dehydrogenase	Formation of NADH Measured at 340 nm	0.26 mg/L
L-Malic acid	L-Malate dehydrogenase Glutamate-oxaloacetate transaminase	Formation of NADH Measured at 340 nm	0.25 mg/L
Malic acid	Malate dehydrogenase	Formation of H_2_O_2_ NADH reduces redox probe Abs measured at 450 nm (WST)	(6.3 μM)**
Oxalic acid	Oxalate oxidase Peroxidase (+ MBTH+ DMAB)	Formation of H_2_O_2_ Indamine dye formed Measured at 590 nm E_590 nm_ = 47,600	NA
Pyruvic acid	D-Lactate dehydrogenase	Consumption of NADH Measured at 340 nm	0.394 mg/L
Pyruvic acid	Pyruvate oxidase Peroxidase	Formation of H_2_O_2_ Peroxidase activated dye Abs measured at 540 nm (AB)	3.0 μM
Succinic acid	Succinyl-CoA synthetase Pyruvate kinase L-Lactate-dehydrogenase	Consumption of NADH Measured at 340 nm	0.256 mg/L

Assays from providers that do not release the enzyme systems used or the nature of the redox or fluorimetric probe used for final detection were not included. Data compiled from the websites of the following providers: Megazyme, Abcam, Cell Biolabs Inc., Trinity Biotech, Libios, and Sciencell. AB, alamarBlue (resazurin); WST, tetrazolium-based dye; MBTH, 3-methyl-2-benzothi-azolinone hydrozone; DMAB, 3-(dimethylamino)benzoic acid. **First standard solution used for calibration in manufacturer booklet. NA, data not available.

## Incorporation Assays (To Monitor Microbial Activity)

The incorporation of labeled or analog substrates in microbial biomass has been widely used (and would certainly deserve a review on its own). The incorporation rate of the selected compounds is assumed to be proportional to the metabolic activity and the viability of anabolically active microbes. As a result, many incorporation assays relying on different detection methodologies have evolved. In addition, many of these techniques are compatible with further use of microscopy and single-cell analysis as well as (meta)genomics and (meta)proteomics tools (not discussed within the scope of this review; see Hatzenpichler et al., 2020, for more information), thus making such incorporation assay very attractive when further functional or taxonomic characterization of a system is considered. The sensitivity of incorporation assays depends on the detection technology on one end, but also the relative amounts of cell component labeled on the other end. In microbial cells, the relative abundance of macromolecules is as follows: proteins [52%–70% dry weight (DW)] > RNA (4%–20% DW) > lipids (8%–9% DW) > DNA (2%–3% DW) (Taymaz-Nikerel et al., 2010; Liao et al., 2011). This makes the choice of the target crucial in order to have the best possible monitoring of the incorporation, depending on the extraction and recovery of the target molecule to be labeled from the sample (or culture). Furthermore, one has to keep in mind that the proportions of these macromolecules might vary a lot depending on the growth conditions and growth rate (Taymaz-Nikerel et al., 2010; Liao et al., 2011).

Overall, the compounds used for incorporation assays fall under three main categories: radioisotopes, stable isotopes, and substrate analogs (Table 3).

**TABLE 3:** Non-exhaustive list of labels commonly used in incorporation assays.

Type of label	Target macromolecule	Detection
**Radioactive**		
3H-thymidine	DNA	Scintillation counter or microautoradiography (MAR)
3H-leucine	Protein	Scintillation counter or microautoradiography (MAR)
14C-leucine	Protein	Scintillation counter or microautoradiography (MAR)
14C-acetate	All	Scintillation counter or microautoradiography (MAR)
14C-glucose	All	Scintillation counter or microautoradiography (MAR)
3H-hypoxanthine	All	Scintillation counter or microautoradiography (MAR)
**Stable isotopes**		
13C	All	Raman, mass spectrometry, density separation
15N	Protein DNA	Raman, mass spectrometry, density separation
2H nucleoside	DNA	Raman, mass spectrometry, density separation
2H (2H2O)	Lipids	Raman, mass spectrometry, density separation
**Substrate analogs**		
L-Azidohomoalanine (AHA)	Protein (methionine analog)	Click chemistry with matching fluorescent or affinity tag
L-Homopropargylglycine (HPG)	Protein (methionine analog)	Click chemistry with matching fluorescent or affinity tag
Ethynyl-D-alanine (EDA)	Protein (alanine analog)	Click chemistry with matching fluorescent or affinity tag
Azido-D-alanine (ADA)	Protein (alanine analog)	Click chemistry with matching fluorescent or affinity tag
*O*-Propargyl-puromycin (OPP)	Protein (alanine analog)	Click chemistry with matching fluorescent or affinity tag
*N*-Azidoacetylmannosamine-tetraacylated (Ac4ManNAz)	Glycosylated protein	Click chemistry with matching fluorescent or affinity tag
*N*-Azidoacetylglucosamine-tetraacylated (Ac4GlcNAz)	Glycosylated protein	Click chemistry with matching fluorescent or affinity tag
*N*-Azidoacetylgalactosamine-tetraacylated (Ac4GalNAz)	Glycosylated protein	Click chemistry with matching fluorescent or affinity tag
5-Bromo-2′-deoxyuridine (BrdU)	DNA (thymidine analog)	Immunostaining or immunocapture
5-Ethynyl-2′-deoxyuridine (EDU)	DNA (thymidine analog)	Click chemistry with matching fluorescent or affinity tag
(2′S)-2′-deoxy-2′-fluoro-5-ethynyluridine (F-ara-EdU)	DNA (thymidine analog)	Click chemistry with matching fluorescent or affinity tag
5-Ethynyl uridine (EU)	RNA (uridine analog)	Click chemistry with matching fluorescent or affinity tag
Alkynyl palmitic acid	Lipids	Click chemistry with matching fluorescent or affinity tag
Azido palmitic acid	Lipids	Click chemistry with matching fluorescent or affinity tag
Alkynyl myristic acid	Lipids	Click chemistry with matching fluorescent or affinity tag
Azido myristic acid	Lipids	Click chemistry with matching fluorescent or affinity tag
Alkynyl stearic acid	Lipids	Click chemistry with matching fluorescent or affinity tag
Azido stearic acid	Lipids	Click chemistry with matching fluorescent or affinity tag

### Radioisotope Incorporation

Radioisotopes are easily detected because of the ionizing radiations they emit. The most commonly used radiolabeled compounds are 3H-thymidine and 3H-leucine or 14C-leucine. These radiolabeled substrates have been used separately or in combination (3H-thymidine and 14C-leucine). Thymidine is incorporated into microbial DNA and thus provides a measure of DNA synthesis. Similarly, leucine is incorporated into proteins and acts as a proxy for protein synthesis. The combination of both allows the measurements of protein synthesis and DNA synthesis in a single experiment. This approach assumes that thymidine and/or leucine is taken up into the cells; however, some microbes, especially strict autotrophs and oligotrophs, lack the necessary transporters for the uptake of many organic molecules, including amino acids and nucleotides (Pérez et al., 2010). Practically, samples are incubated with radiolabeled substrates at a final concentration within the μM range (from 1 μm for thymidine to 15 μM for leucine; see an example in Tuominen, 1995), ensuring saturation (Bååth, 1998; Bloem and Bolhuis, 2009). It must be noted that such concentrations are at least two to four orders of magnitude higher than the concentrations of these compounds in the environment, where they typically do not exceed the nM range (Jørgensen, 1982, 1987). This certainly has an effect and must be considered as a potential limitation of such approach. The incubation is usually short to avoid inducing changes in the microbial community structure and potential deleterious effects of ionizing radiation and ranges from a few minutes (Tuominen, 1995) to a few hours (Bååth, 1998; Bloem and Bolhuis, 2009) depending on the nature and activity of the sample. After incubation, the incorporation is stopped by cooling and/or adding ethanol or trichloroacetic acid (TCA). Labeled protein or DNA is then extracted and precipitated, taking care to remove unincorporated radiolabeled thymidine and leucine. Finally, after a final solubilization step, labeled macromolecules are determined using a scintillation counter and the incorporation rate can be calculated. The use of radiolabeled isotopes is becoming less frequent as less demanding alternatives (in terms of labor, safety, and waste management) are developed (see following sections). Still, other radioactive substrates are still in use for specific purposes such as in parasitology (3H hypoxanthine) and environmental science (14C acetate, 14C glucose) in combination with microautoradiography (MAR) (Brun and Kunz, 1989; Lee et al., 1999; Chidthaisong and Conrad, 2000; Nielsen et al., 2003a; Maerki et al., 2006).

### Stable Isotope Probing

Stable isotope probing (SIP) refers to the use of stable isotope-labeled substrates to probe the microbial utilization of these specific substrates to build their macromolecules (Jehmlich et al., 2010; Emerson et al., 2017; Hatzenpichler et al., 2020). As a result, DNA-, RNA-, phospholipid fatty acid (PLFA)-, and protein-SIP are possible using different stable isotopes (mostly 2H, 13C, 15N, and 18O). Comparing the different SIP targets, one can see a trade-off between the amount of incorporation required, the workload, and the taxonomic information gained (Jehmlich et al., 2010). Indeed, protein-SIP can provide some taxonomic information; however, the gold standard remains DNA-SIP. In most studies, the use of SIP is intended to gather taxonomic or proteomic information about the anabolically active part of the microbial population. Much valuable information on metabolic rates can be gathered in the analytical process as well. Most SIP studies rely on the extraction of target macromolecules (i.e., DNA, RNA, phospholipids, and protein) and further analysis using liquid chromatography coupled with MS. In this context, several indicators gathered from MS data have been recognized as useful proxies for metabolic rates and carbon fluxes. In particular, labeling ratio (lr), relative isotope abundance (RIA), and the shape of the isotope pattern of specific peptides can provide valuable information (Jehmlich et al., 2010; Taubert et al., 2011; Von Bergen et al., 2013). The lr is the ratio of the intensity of the isotope pattern of labeled peptide and the total intensity of isotope patterns of unlabeled and labeled peptides. This ratio is considered to be an indicator of protein synthesis and turnover in time-resolved analysis. Indeed, the evolution of the lr over time closely matches the growth curve for protein expressed under growth conditions (Taubert et al., 2011; Von Bergen et al., 2013) and can be processed using growth curve fitting equations. For such analysis, the choice of the protein or peptides analyzed is therefore crucial, and several peptides might be analyzed. RIA is the percentage of stable isotope incorporation estimated using the extent of mass shift of the peptide peak. It is a proxy for the metabolization of carbon (or nitrogen) (Von Bergen et al., 2013). Finally, an ideal Gaussian distribution of the m/z (mass over charge) values of a peptide indicates direct substrate utilization. On the other hand, m/z value distribution for a peptide showing an isotope pattern with negative skewness (i.e., differing from the ideal Gaussian distribution and tailing toward lower m/z values) indicates indirect 13C substrate metabolization (e.g., through cross-feeding) (Von Bergen et al., 2013). These three indicators have been investigated with protein-SIP until now; however, one can assume that they can be used with PLFA-SIP as well.

As an alternative to MS, Raman microspectroscopy can be used in combination with optical tweezers or single-cell ejection to sort and separate labeled from unlabeled cells with high accuracy. The incorporation of heavy isotopes in the cell macromolecules results in a shift of the Raman peaks toward lower wavenumbers compared with unlabeled cell spectra (Von Bergen et al., 2013; Wang et al., 2013; Jing et al., 2018). That detectable Raman shift can be used to manually or automatically trigger the use of optical tweezers or single-cell ejection system to allow rapid screening of cells and keep them for further analysis (metagenomics) and eventual metabolic profiling (Von Bergen et al., 2013; Wang et al., 2013; Jing et al., 2018; Lee et al., 2019). In this context, Raman spectra can be used directly to monitor the incorporation rate, as the shift observed is proportional to the incorporation of heavy isotopes and can be from the bulk biomass (Li et al., 2013) down to the single-cell level using spontaneous Raman microspectroscopy (Wang et al., 2013; Jing et al., 2018; Lee et al., 2019) or surface-enhanced Raman spectroscopy (SERS) (Wang Y. et al., 2016).

### Substrate Analog Probing

Substrate analog probing is a term introduced by Hatzenpichler (Hatzenpichler et al., 2020) to differentiate the approach from SIP. This approach uses synthetic compounds that are structurally and/or functionally analogous to natural molecules. Such analogs are incorporated into cell macromolecules due to enzyme promiscuity. Some of these substrate analogs already bear a fluorescent tag, allowing their direct detection; however, most analogs must be detected after incorporation using immunocapture, immunostaining, or “click chemistry” (i.e., azide–alkyne cycloaddition; Liang and Astruc, 2011) reactions with an appropriate fluorescent or affinity tag. The variety of substrate analogs available nowadays allows monitoring of the incorporation of substrate analogs into all types of macromolecules (i.e., DNA, RNA, protein, and lipids; Table 3).

Similarly to radiolabeled thymidine incorporation, 5-bromo-2′-deoxyuridine (BrdU) has been used in the same way. Indeed, BrdU is an analog of the DNA precursor thymidine. When it is added to a sample containing growing cells (culture or environmental sample), the cells incorporate BrdU into their DNA instead of thymidine. The BrdU-containing DNA is then detected using an anti-BrdU antibody. Then, a secondary antibody specific for the anti-BrdU primary antibody is used to capture the DNA (with the help of paramagnetic beads), thus providing a new sample of reduced complexity that corresponds to cells effectively dividing (Men et al., 2011; Robbins et al., 2011), which can be further analyzed using molecular methods (Robbins et al., 2011). Alternatively, for visualization or quantification purposes, the secondary antibody can be coupled with a fluorescent tag. As for other incorporation assays, one can assume that the incorporation rate is proportional to the metabolic activity. However, it must be noted that in some biofilms or when experiencing limitation in nitrogen or phosphorus availability, bacteria can show high metabolic activity but poor growth and thus very low DNA synthesis. In addition, it was shown that many species are not able to incorporate BrdU and that interpretation should be done with care when using BrdU. Urbach et al. (1999) stated that “while BrdU incorporation can be used to prove that specific populations of bacteria in a natural ecosystem are growing, this method cannot be used conversely to prove that a population is not growing, unless it is also demonstrated that the species in question can assimilate BrdU.”

**FIGURE 10:**
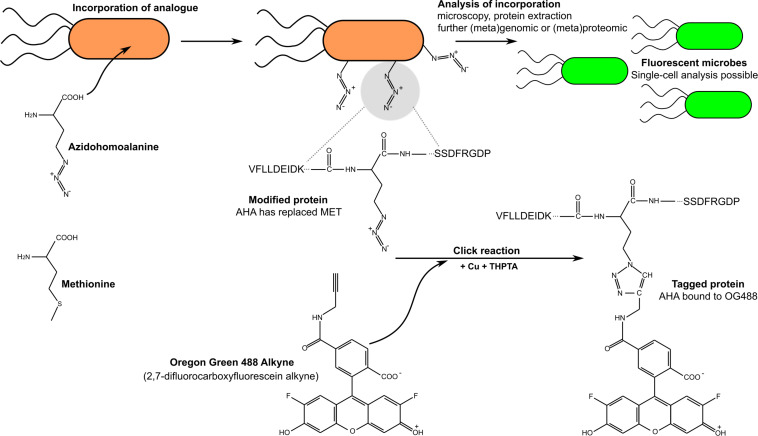
Simplified sketch of bio-orthogonal noncanonical amino acid tagging (BONCAT) procedure using azido-alanine and the following click chemistry reaction with matching Oregon Green 488 alkyne fluorescent probe. Note that for simplicity, the sketch only shows extracellular azidohomoalanine (AHA); however, after the click reaction, both intracellular and extracellular AHA-labeled peptides are detected.

Among all the methods developed, bio-orthogonal noncanonical amino acid tagging (BONCAT) has garnered much interest because of its compatibility with many other approaches such as FACS, fluorescence *in situ* hybridization (FISH), nanoscale secondary ion mass spectrometry (nanoSIMS), (meta)genomics, and (meta)proteomics (Landgraf et al., 2015; Hatzenpichler et al., 2020). In BONCAT, a methionine analog such as azide-bearing azidohomoalanine (AHA) or alkyne-bearing homopropargylglycine (HPG; syn: 2-amino-5-hexynoic acid) is used to label newly formed proteins (Landgraf et al., 2015). Indeed, methionine-tRNA ligase (EC: 6.1.1.10: syn methionyl-tRNA synthetase) can accommodate amino acid analogs such as homopropargylglycine (HPG) and AHA but at a slower rate. Between these two methionine analogs, HPG incorporation is still slower than AHA incorporation (Kiick et al., 2002; Sherratt et al., 2017). After an incubation step (preferably in a medium without methionine), the cells are usually fixed to increase sample stability during storage and then labeled using click chemistry (Figure 10) using the matching azide or alkyne tag. A variety of fluorescent tags have been developed and used in combinations with FACS and microscopy to detect and/or sort anabolically active cells (Singer et al., 2017).

Similarly, biotin tags allowing quantification, affinity purification, and further identification of protein using MS have been used as well (Landgraf et al., 2015). Although BONCAT is intended more for further genomic or proteomic characterization, labeling rates of cells or specific proteins can also be used as a proxy for metabolic rates (Hatzenpichler et al., 2014). Indeed, the evolution of fluorescence of a culture or from extracted proteins matches 3H-leucine incorporation (Leizeaga et al., 2017).

## Isothermal Calorimetry

In living organisms, the result of metabolic activity leading to maintenance, development, and reproduction also includes some heat release (Antoine and Laplace, 1921; Von Stockar, 2010, 2013). The accompanying enthalpy changes can be measured using calorimetry (Russel et al., 2009) and especially isothermal microcalorimetry. Most of the recent isothermal microcalorimeters are heat conduction calorimeters. In such instruments, metabolic heat produced in a sealed calorimeter vial is allowed to flow to a thermostatted heat sink that has very high heat capacity (usually 100 times higher than that of the sample). The thermoelectric module placed between the sample and the heat sink allows minute temperature differences between the two to be converted into electric signals that can be easily recorded and calibrated to be proportional to the heat flow (Wadsö, 2002; van Herwaarden and Iervolino, 2014). The heat flow (or thermal power), expressed in W, is directly proportional to the metabolic activity (measured by other means such as oxygen consumption rate; for example, Brueckner et al., 2016). The passive and external measurement (through the thermopile placed below vials that are kept sealed) makes isothermal microcalorimetry well suited for measuring metabolic activity in general, but even more advantageous for solid and opaque samples (such as blood, soil, sediment, milk, and agar, for example; Alklint et al., 2005; Rong et al., 2007; Trampuz et al., 2007; Krišèiunaite et al., 2011; Nielsen et al., 2017).

In addition, isothermal calorimetry is a quantitative tool, and for some types of metabolism and media, the enthalpy of the overall metabolic reaction (ΔH_rxn_) can be easily estimated (for example, when biomass formation can be neglected; see Bravo et al., 2011; Bravo et al., 2014). This allows direct calculation of the reaction rate from the heat dissipation rate (i.e., the heat flow). Furthermore, coupling metabolic reaction with CO_2_ measurement directly within the calorimeter allows rapid additional insight into the metabolic rates and yield, for example. In this context, many authors have demonstrated that enthalpy change and CO_2_ production can be determined simultaneously by many calorimetry instruments using a CO_2_ trap containing NaOH 1 M (usually an HPLC insert) and measuring the additional enthalpy caused by the hydration of CO_2_ into HCO_3_^–^ (–108.5 kJ⋅mol^–1^) (Criddle et al., 1991; Hansen et al., 2004; Barros et al., 2010), thus helping to rapidly match CO_2_ production and heat dissipation. This approach is referred to as calorespirometry and can be very useful in determining biomass formation or yield using a few more pieces of data (see next section).

## Relations and Correlations Between Assays

As all of these techniques have the same goal (i.e., measuring metabolism), the consequence is that many of them provide data that can be correlated or combined (Nocker et al., 2011). Indeed, several studies have correlated two or more of the abovementioned assays for microbes (Cooney et al., 1969; Rodriguez et al., 2011; Braissant et al., 2015) or higher organisms (Braeckman et al., 2002). This is important, as it allows rapid validation of the use of a technique or deciphering between different types of metabolism. In this context, a lack of correlation might sometimes be more informative than correlation itself. For example, high activity measured by an assay without matching electron acceptor reduction might point to the use of an alternative electron acceptor (NO_3_^–^, for example) or fermentation (*sensu stricto*). Similarly, calorespirometric ratios (heat per O_2_ and heat per CO_2_) can give valuable information. Heat per O_2_ can help detect the presence of anaerobic processes, and heat per CO_2_ can provide hints on the type of carbon source used (carbohydrate, protein, or lipids) (Hansen et al., 2004). For heat per O_2_, the Thornton rule states that the enthalpy of combustion of organic compounds is roughly constant when expressed per mole of O_2_. The average value of this ratio is −455 ± 15 kJ⋅mol^–1^ O_2_; that is, −107 to −120 kJ⋅mol^–1^ e- (Thornton, 1917; Hansen et al., 2004; Maskow et al., 2010; Von Stockar, 2013). Therefore, for respiring organisms (and irrespective of the carbon source), the ratio can be used to estimate these parameters (carbon source consumption or oxygen consumption). More importantly, deviation from the oxycaloric ratio can provide information on the presence of anaerobic processes and estimation of the fraction of anaerobic metabolism at steady state. Similarly, heat per CO_2_ is a proxy for efficiency, as it is an approximate measure of the rate of catabolism over the rates of catabolism plus anabolism. Low values of heat per CO_2_ indicate that only little energy is lost from catabolism (Hansen et al., 2004; Wadsö and Hansen, 2015). Still, care must be taken when using such ratios, as the presence or use of nitrogen-containing compounds (such as urea) and peroxides might lead to significant deviation from the Thornton rule (Hansen et al., 2004; Wadsö and Hansen, 2015). In addition, calorespirometric ratios overall for many types of metabolism, even more complex types combining several of these assays, can provide a rather complete metabolic budget (carbon, nitrogen, oxygen, energy). An example for glucose respiration is given below:

aCH6O12+6bO+2cNH→4+CHO1.8N0.5+0.2dCO+2eHO2+fH+

The following conservation equations can now be written:

(note that for the calculations below, *d*, *e*, and *f* are negative)

C conservation: 6*a* + 1 + *d* = 0

H conservation: 12*a* + 4*c* + 1.8 + 2*e* + *f* = 0

O conservation: 6*a* + 2*b* + 0.5 + 2*d* + *e* = 0

N conservation: *c* + 0.2 = 0

Charge conservation: *c* + *f* = 0

As these equations clearly show, all variables are not independent, and solving for only a few of the parameters (*a, b, c, d, e*, and *f*) allows for determining the other ones. Moreover, calorimetry can provide an additional equation to solve this system. Indeed, using the enthalpy of reaction provides an additional equation that can further help solve the equation system. The enthalpy of reaction (ΔH_rxn_) measured by the calorimeter can also be calculated using enthalpy of formation (ΔH_f_) or of combustion (ΔH_c_); using enthalpy of combustion simplifies the equation even more (see details in Maskow and Harms, 2006; Barros et al., 2010; Maskow et al., 2010).

ΔH_rxn_ = −(ΔH_c_ C_biomass_ + *f⋅*ΔH_c_ H − *a⋅*ΔH_c_ C_glc_ − *c⋅*ΔH_c_ H_NH4_) (from Maskow et al., 2010)

The enthalpies of formation and combustion of organic matter, and especially organic matter from microbial biomass, can be found in several review papers (Cordier et al., 1987; von Stockar et al., 1993; Popovic, 2019), thus making this approach easy to implement with relatively high accuracy.

However, such an approach requires that the metabolism of the organism (or group of organisms) is already known and that the organisms have been characterized up to the point where the growth equation can be written in an accurate manner.

## Future Perspectives

Many of these assays have been used for a long time and have been improved and optimized using new chemicals and instruments. Still, as mentioned for glucose, many studies continue to improve or adapt (not to say “hack” or “exploit”) these methods and approaches. Among the possible promising approaches for many of these assays, the lab on a chip is very appealing. Microfluidic systems, piezoelectric pumps, and sub-millimeter size sensors make it possible to build an array of sensors that can monitor different compounds over time and thus provide real-time monitoring of metabolic rates. It is likely that, in the future, many of these assays (or replacements for some of them) will be combined on single disposable chips. Alternatively, for now, 3D printing and rapid prototyping methods also allow rapid development of microfluidic devices that can incorporate such new sensors. Finally, it must be noted that such chips are very close to becoming reality, as several authors have already presented multiple parameters using separate microprobes or microfluidic chips simultaneously measuring pH, OD, and dissolved oxygen, for example (Wong et al., 2015; Guimer et al., 2019). Still, as this is a rapidly evolving field, many new sensor types might appear in the next years.

## Author Contributions

OB and MA-F drafted and wrote the manuscript. TW and GB critically reviewed the manuscript and improved it. All authors read and approved the submitted version.

## Conflict of Interest

GB was employed by the company Alta-Uro AG. The remaining authors declare that the research was conducted in the absence of any commercial or financial relationships that could be construed as a potential conflict of interest.

## Footnotes

1 https://bionumbers.hms.harvard.edu/

2 https://www.unisense.com/MicroOptode
